# Craig’s Microscope

**Published:** 1862-07

**Authors:** 


*Craig's Microscope.*—We have received from the inventor this little optical instrument, patented February 18, 1862. It has high magnifying power, and shows many objects remarkably well. It is exceedingly simple in construction, requiring no time for adjustment, and shows with some good degree of distinctness, blood corpuscles, milk globules, cancer cells, as well as the animalculæ of stagnant water. It seems quite remarkable that so simple an instrument, costing but $2.25, should show so much, and show it so well.

